# The effect of FTO rs9939609 polymorphism on the association between colorectal cancer and dietary fiber

**DOI:** 10.3389/fnut.2022.891819

**Published:** 2022-10-03

**Authors:** Soroor Fathi, Mina Ahmadzadeh, Mahsa Vahdat, Maryam Afsharfar, Zahra Roumi, Naeemeh Hassanpour Ardekanizadeh, Soheila Shekari, Seyed Mohammad Poorhosseini, Maryam Gholamalizadeh, Sepideh Abdollahi, Elham Kheyrani, Saeid Doaei

**Affiliations:** ^1^Department of Community Nutrition, School of Nutrition and Food Science, Isfahan University of Medical Sciences, Isfahan, Iran; ^2^Department of Clinical Nutrition and Dietetics, Faculty of Nutrition and Food Technology, National Nutrition and Food Technology Research Institute, Shahid Beheshti University of Medical Sciences, Tehran, Iran; ^3^Aboozar Children's Medical Center, Ahvaz Jundishapur University of Medical Sciences, Ahvaz, Iran; ^4^Department of Nutrition, School of Medicine, Zahedan University of Medical Sciences, Zahedan, Iran; ^5^Master of Science Student of Department of Nutrition, Science and Research Branch, Islamic Azad University, Tehran, Iran; ^6^Torbat Jam Faculty of Medical Sciences, Torbat Jam, Iran; ^7^Department of Nutrition, Science and Research Branch, Islamic Azad University, Tehran, Iran; ^8^Genomic Research Center, Department of Medical Genetic, Shahid Beheshti University of Medical Sciences, Tehran, Iran; ^9^Cancer Research Center, Shahid Beheshti University of Medical Sciences, Tehran, Iran; ^10^Department of Medical Genetics, School of Medicine, Tehran University of Medical Sciences, Tehran, Iran; ^11^Taban Medical Genetic Laboratory, Tehran, Iran; ^12^Research Center of Health and Environment, School of Health, Guilan University of Medical Sciences, Rasht, Iran; ^13^Department of Community Nutrition, School of Nutrition and Food Sciences, Shahid Beheshti University of Medical Sciences, Tehran, Iran

**Keywords:** colorectal cancer, FTO gene, dietary fiber, polymorphism, genotype

## Abstract

**Background:**

Gene polymorphisms may explain the controversy on the association between colorectal cancer (CRC) and dietary fibers. The purpose of this study was to investigate the effect of fat mass and obesity-associated (FTO) rs9939609 polymorphism on the association between colorectal cancer and dietary fiber.

**Methods:**

This case-control study was conducted on 160 CRC cases and 320 healthy controls in Tehran, Iran. The participants' food intake was assessed using a semi-quantitative food frequency questionnaire (FFQ). The frequency of rs9939609 FTO polymorphism in the case and control groups was determined using the tetra-primer amplification refractory mutation (tetra-ARMS) method.

**Results:**

In the participants with the TT genotype of the FTO rs9939609, the cases had higher BMI and lower intake of dietary fiber compared to the controls (*P* = 0.01). Among A allele carriers of FTO rs9939609 polymorphism, the cases had higher BMI (*P* = 0.04) and lower intake of total fiber (*P* = 0.02) and soluble fiber (*P* = 0.02). An inverse association was found between CRC and dietary fiber intake among those with the AA/AT FTO rs9939609 genotype after adjusting for age, sex, smoking, alcohol consumption, physical activity, BMI, and calorie intake (OR = 0.9, CI 95%:0.84–0.92, *P* < 0.05).

**Conclusion:**

This study found a link between higher dietary fiber consumption and a lower risk of CRC in A-allele carriers of FTO rs9939609 polymorphism. Future studies are needed to identify the underlying mechanisms of the association between CRC and dietary fibers in people with different FTO genotypes.

## Introduction

Colorectal cancer (CRC) is the second most common cancer among women after breast cancer and the third most common cancer among men after lung and prostate cancer worldwide (1). The incidence of CRC is high in developed countries, and there are rapid increases in CRC cases in developing countries such as Iran. Based on a screening program completed in 2018, there was an increasing trend in the risk of CRC among the Iranian people and 33 Iranians per 100,000 had CRC (2).

The pathophysiology of CRC includes a complex interaction of genetics and environmental factors. Westernized dietary habits, high consumption of refined sugars and animal fats, low consumption of fruits and vegetables, consumption of tobacco and alcohol, obesity, and low physical activity are the main environmental risk factors of CRC (3–7). Dietary fiber is a non-digestible carbohydrate, mainly found in fruits and vegetables. The association between dietary fiber and the risk of CRC is not yet clear. Dietary fiber may plays an essential role in preventing CRC through several mechanisms such as improving serum glycemic and lipid profile (8, 9), increasing stool bulk, diluting fecal carcinogens, and decreasing transit time of foods in gastrointestinal tract. A previous study found that doubling dietary fiber intake in populations with low fiber intake reduced the risk of CRC by up to 40% (10). However, a pooled analysis of prospective cohort studies found that high dietary fiber intake was not associated with a reduced risk of colorectal cancer (11).

On the other hand, obesity was identified as an independent risk factor for CRC across all age groups (12). The fat mass and obesity-associated (FTO) gene polymorphisms may increase food intake, probably leading to a positive energy balance and obesity (13). Several studies have reported a strong association of the FTO polymorphisms with the risk of CRC. However, one recent meta-analysis failed to find any association between CRC and FTO genotype (14). CRC may be associated with the FTO gene only in people with a specific diet (15). Furthermore, the link between CRC and dietary components such as fiber may be affected by obesity-related genes (13). Thus, this study aimed to investigate the effect of FTO genotype on the association between CRC and dietary fiber.

## Methods

This hospital-based case-control study was performed on 480 participants, including 160 patients with CRC as a case group and 320 healthy individuals as a control group. A 1:2 case-to-control ratio was used in this matched case-control study due to concern for sufficient numbers in stratified analysis and increase in power given the expected prevalence of exposure among the controls. The required sample size was estimated using OpenEPI online software (http://www.openepi.com/SampleSize/SSCC.htm) according to a previous similar study (16). The required samples were randomly selected from Firoozgar hospital in Tehran, Iran from June 2020 to June 2021. Patients had histologically confirmed stage 3 and 4 CRC and underwent adjuvant chemotherapy with FOLFOX4 (5-fluorouracil, leucovorin and oxaliplatin) after surgery. Due to the fact that all the patients were recruited from one hospital and all of them were on the same stage of the disease and underwent surgery, they were treated with the same drugs. The control group was randomly selected among non-cancer patients referring to Firoozgar hospital for general check-up. All controls had undergone a colonoscopy within recent 2 years of participating to confirm the absence of colon cancer. Inclusion criteria of the case group included the willingness to participate in this study, histological confirmation of CRC in individuals, maximum of 2 months passing from cancer diagnosis, no other diseases affecting food intake, and the age range of 35 to 70 years. Inclusion criteria for the healthy control group were the willingness to participate in this study, no history of malignant diseases, no disease affecting food intake, and an age range of 35 to 70 years. All participants were informed of the study details, and written consent was obtained before the data collection. Those who were unable to answer the researchers' questions about the information required for the project were excluded from the study (*n* = 51). The final analyses were performed on 429 participants including 135 cases and 294 controls.

Data on socio-demographic factors and the medical history of individuals was collected through a face-to-face interview. Also, all participants were evaluated for anthropometric status, dietary intake, and physical activity. FTO gene profiles were also obtained by collecting blood samples. Details on how to collect data are provided below.

### Assessment of socio-demographic status and physical activity

Information related to the demographic, economic, and social level of the participants including age, sex, education, income level, marital status, health status, smoking status, and alcohol consumption was collected separately through general information questionnaires. Also, information about the patients' medical history including diabetes, inflammatory bowel disease, and family history of CRC in first degree relatives (father, mother, child, brother, and sister) and second degree (grandfather, grandmother, uncle, aunt, and grandson) was collected.

The level of physical activity was assessed using the International Physical Activity Questionnaire, which has already been validated in Iran (17). The IPAQ collects PA data by asking participants to recall their vigorous and moderate activities during the last 7 days (18). Using this questionnaire, the activity level of people at home, during exercise, during travel, and in sitting activities was determined. Individuals were evaluated and compared in terms of activity using the metabolic rate of activity (MET).

### Anthropometric measurements

The participants' height and weight were measured using a standard stadiometer with an accuracy of 0.5 cm and a calibrated Seca scale (Seca, Hamburg, Germany) with an accuracy of 100 g, respectively. Then, body mass index (BMI) was calculated as bodyweight divided by height squared (kg/m^2^).

### Assessment of fiber intake

The food intake of participants in the study was assessed by completing a 168-items semi-quantitative food frequency questionnaire (FFQ) that has already been validated in Iran (19). The FFQ is commonly used in nutritional epidemiology to examine dietary intakes. Information about the intake of nutrients during the year before the diagnosis was collected through a face-to-face interview by a trained dietitian. All data obtained from FFQ was converted to grams and were analyzed using Nutritionist IV software (First Databank, the Hearst Corporation, San Bruno, CA, United States) which was modified for Iranian foods. The intake of different types of dietary fiber including soluble fiber, insoluble fiber, and crude fiber was assessed.

### FTO genotyping

For genetic assessment, 5 ml of blood samples were taken from all participants in EDTA tubes (EDTA K3, Shandong Weigao Group Medical Polymer Co., Ltd, China). The samples were transferred to Taban Laboratory in Tehran, Iran. The sample was stored in a freezer at−20 °C until the experiments.

Samples were washed with (Tris-HCl 10 mmol / L, MgCl2 5 mmol/L, Lysis Buffer Triton X 1% pH 7.6, and Phosphate Buffer (PBS). Next saline buffer and red blood cells (RBCs) were removed from the medium, followed by salting out DNA from white blood cells (WBCs). The primers required to perform the PCR reaction were designed by extracting the sequence of adjacent SNP regions. In addition, appropriate primers were designed using Primer1 software by referring to the dbSNP database located at http://www.ncbi.nlm.nih.gov/SNP. The desired SNP was genotyped by allele-specific primers according to the last nucleotide at the end of the '3 primer and complement of the SNP site, using Tetra-primer ARMS PCR. This technique required four primers to genetically amplify a large fragment of template DNA containing the SNP (control fragment) and two smaller fragments representing each of the two allele products (Table 1). The primers were designed to produce allelic amplified fragments of different sizes and to be distinguishable by agarose gel electrophoresis. In addition to the first non-pairing at the end of the 3' internal specific primers, the reaction specificity was increased by creating an additional pairing at the third position of the 3' end of each of the two internal specific primers. For specific amplification of fragments around the desired polymorphism, polymerase chain reaction (PCR) in a volume of 25 μl including 200 ng DNA, 3 μl 10X PCR buffer, 5/1 μl dNTP 10 mmol, 1 μl MgCl250 mmol, 5/1 picomol of Rivers external primer and forward Internal OR) and (IF, 1 picomole of internal Rivers primer)(IR, 5/0 picomole of external input primer)(OF, 4/0 μl (2 units) of DNA Taq polymerase (CinnaGen Research & production Co., Alborz, Iran) was performed by Eppendorf thermocycler PCR, Germany. In the next step, PCR products were electrophoresed on agarose 1% gel for 40 min at 80 volts. Finally, the separated bands were observed and analyzed by UV Gel Documentation. After determining the genotype of individuals, the association between cancer and genotype was calculated using the odds ratio (OR) method.

**Table 1:** Sequence and characteristics of primers designed for PCR and identification of rs9939609 FTO gene polymorphism.

**Gene**	**Primer sequence**	**Product size**	**Annealing temp. °C**
**FTO**	Forward outer primer: AGTTCCAGTCATTTTTGACAGC Reverse outer primer: AGCCTCTCTACCATCTTATGTC Forward inner primer: CCTTGCGACTGCTGTGAATATA Reverse inner primer: GAGACTATCCAAGTGCATCTCA	Control fragment: 429bp T allele: 278bp A allele: 194bp	59.5

### Statistical analysis

The Hardy-Weinberg equilibrium was used to evaluate the genotype distribution. General characteristics and frequency of rs9939609 FTO polymorphism in case and control groups were compared by Chi-square (for qualitative variables) and independent *t*-test (for quantitative variables). Analyses were performed based on the Dominant genetic model (AA + AT vs. TT).

Chi-square (for qualitative variables) and independent *t*-test (for quantitative variables) methods were used to compare socio-demographic indicators and dietary intake in case and control groups with different genotypes of the FTO gene. The relationship between dietary fiber and CRC in individuals with different genotypes of rs9939609 polymorphism of FTO gene was performed by dual logistic regression method. In addition, the regression models to investigate the relationship between CRC and risk allele based on the Dominant genetic model (TT vs. AT + AA) in raw form (model 1), after adjusting age and sex (model 2), adjusting age, sex, smoking, alcohol consumption and physical activity (model 3), and adjusting age, sex, smoking, alcohol consumption, and physical activity and BMI (model 4) were assessed. Statistical analysis was performed using SPSS software version 20 (SPSS Inc., Chicago, United States), and *P*-value <0.05 was considered statistically significant in all analyses.

## Results

The general characteristics of the participants based on the dominant model of FTO rs9939609 polymorphism (TT vs. AA/AT) are presented in Table 2. Among the participants with TT genotype of FTO rs9939609 polymorphism, the cases had higher BMI (29.18 ± 3.9 vs. 27.67 ± 3.31 kg/m^2^, *P* = 0.02) than the controls. Among A-allele carriers of FTO rs9939609 polymorphism, the cases had higher age (52.5 ± 17.19 vs. 48.07 ± 11.19 y, *p* = 0.001), smoking (42% vs. 13%, *P* = 0.005), bodyweight (70.03 ± 10.89 vs. 69.62 ± 9.08 kg, *P* = 0.03), and BMI (28.68 ± 4.06 vs. 27.58 ± 3.25 kg/m^2^, *p* = 0.04), and lower height (156.2 ± 5.71 vs. 158.65 ± 7.81 kg, *p* = 0.02) compared to the controls. The minor allele frequency (MAF) was 0.41 in the case group and 0.33 in the control group. Also, the overall minor allele frequency was 0.36.

**Table 2:** General characteristics of the case and control groups based on FTO rs9939609 genotype.

	**TT genotype (*****n*** = **154)**			**AA/AT genotype (*****n*** = **275)**		
	**Cases** ** (*n* = 46)**	**Controls** ** (*n* = 108)**	***P***	**Cases** ** (*n* = 89)**	**Controls** ** (*n* = 186)**	***P***
Age (years)	51.22 ± 16.08	47.37 ± 10.7	0.14	52.5 ± 17.19	48.07 ± 11.19	0.001
Gender
Males	25 (54.7%)	41 (47.6%)	0.08	58 (64.6%)	106 (57.3%)	0.2
Females	21 (45.3%)	57 (52.4%)		31 (35.4%)	80 (42.7%)	
Alcohol use *N* (%)	15 (33%)	5 (5%)	0.06	9 (10.5%)	14 (7.6%)	0.46
Alcohol use *N* (%)	15 (33%)	5 (5%)	0.06	9 (10.5%)	14 (7.6%)	0.46
Smoking *N* (%)	3 (6%)	9 (8.6%)	0.59	37 (42%)	24 (13%)	0.005
Bodyweight (kg)	70.85 ± 10.26	68.82 ± 7.86	0.18	70.03 ± 10.89	69.62 ± 9.08	0.03
Height (cm)	155.83 ± 4.72	157.83 ± 6.87	0.08	156.2 ± 5.71	158.65 ± 7.81	0.02
BMI (kg/m2)	29.18 ± 3.9	27.67 ± 3.31	0.02	28.68 ± 4.06	27.58 ± 3.25	0.04
Physical activity (hrs/wk)	7.37 ± 1.84	7.30 ± 1.64	0.83	7.61 ± 2.02	7.33 ± 1.54	0.11

Regarding to dietary intake (Table 3), among the participants with TT genotype of FTO rs9939609 polymorphism, the cases had higher intake of calorie, protein, carbohydrate, fat, total fiber, soluble fiber, and lower intake of insoluble fiber, and crude fiber compared to the controls. There was no significant difference between the two groups in terms of micronutrients intake. Among A-allele carriers of FTO rs9939609 polymorphism, the cases had higher calorie intake, protein, carbohydrate, fat, and lower intake of total fiber, soluble fiber, insoluble fiber, and crude fiber.

**Table 3:** Dietary intake of the case and control groups based on FTO rs9939609 genotype.

	**TT genotype**			**AA/AT genotype**		
	**Cases**	**Controls**	***P***	**Cases**	**Controls**	***P***
Calorie (kcal/d)	2,594.64 ± 333.4	2,500.48 ± 165.87	0.02	2,555.62 ± 449.78	2,491.33 ± 182.2	0.46
Protein (g/d)	86.23 ± 13.6	86.14 ± 7.85	0.98	85.16 ± 23.3	85.43 ± 9.75	0.68
CHO (g/d)	369.47 ± 53.39	361.08 ± 28.88	0.21	367.88 ± 48.7	351.62 ± 34.95	0.95
Fat (g/d)	93.25 ± 17.13	86.57 ± 10.38	< 0.001	89.84 ± 22.26	90.03 ± 10.64	0.17
Soluble fiber (g/d)	1.17 ± 0.65	1.16 ± 0.81	0.92	0.9 ± 0.65	1.02 ± 0.78	0.02
Soluble fiber (g/d)	1.17 ± 0.65	1.16 ± 0.81	0.92	0.9 ± 0.65	1.02 ± 0.78	0.02
Insoluble fiber (g/d)	5.28 ± 1.99	5.47 ± 2.71	0.63	4.91 ± 1.67	5.3 ± 2.23	0.06
Crude fiber (g/d)	9.17 ± 2.21	10.04 ± 3.58	0.07	9.08 ± 2.53	9.7 ± 3.41	0.40
Total fiber (g/d)	23.99 ± 4.42	26.41 ± 7.63	0.01	23.77 ± 5.02	25.67 ± 4.62	0.02
Iron (mg/d)	18.49 ± 2.69	18.86 ± 3.44	0.49	18.69 ± 2.9	18.54 ± 2.67	0.66
Calcium (mg/d)	1,226.31 ± 53.03	1,195.58 ± 140.29	0.07	1,237.33 ± 228.90	1,234.19 ± 622.43	0.95
Magnesium (mg/d)	335.79 ± 32.86	330.70 ± 47.20	0.47	350 ± 96 ± 65.54	346.80 ± 71.20	0.61
Phosphorus (mg/d)	1,403.16 ± 99.28	1,391.25 ± 201.31	0.65	1,422.52 ± 243.33	1,411.49 ± 563.60	0.82
Zinc (mg/d)	10.50 ± 2.51	10.86 ± 2.42	0.42	10.70 ± 3.17	10.53 ± 3.62	0.67
Copper (mg/d)	1.49 ± 0.65	1.61 ± 0.68	0.32	1.76 ± 0.71	1.64 ± 0.67	0.15
Manganese (mg/d)	5.19 ± 1.95	4.67 ± 1.62	0.13	5.18 ± 1.65	5.20 ± 1.65	0.91
Selenium (mcg/d)	56.50 ± 22.97	49.74 ± 22.83	0.15	67.26 ± 15.11	68.41 ± 16.53	0.54
Fluoride (mg/d)	13,967.59 ± 5,662.25	16,708.70 ± 11,263.98	0.06	11,112.32 ± 3,051.44	10,806.85 ± 3,489.56	0.43
Chromium (mcg/d)	0.13 ± 0.18	0.12 ± 0.17	0.84	0.10 ± 0.15	0.09 ± 0.15	0.65
Iodine (mcg/d)	0.00	0.00		0.00	0.00	
Molybdenum (mcg/d)	50.81 ± 7.45	50.93 ± 10.29	0.94	50.42 ± 4.72	50.88 ± 4.72	0.43
Vitamin A (mcg/d)	819.70 ± 251.03	850.94 ± 307.34	0.53	712.76 ± 113.86	688.77 ± 172.60	0.15
Beta-carotene (mg/d)	2,189.73 ± 474.30	2,034.09 ± 623.76	0.11	2,461.75 ± 772.57	2,482.07 ± 1,058.26	0.85
Vitamin E (mg/d)	10.58 ± 1.14	10.00 ± 4.15	0.45	13.99 ± 6.40	12.63 ± 4.66	0.06
A-tocopherol (mg/d)	8.46 ± 2.91	9.54 ± 5.81	0.15	9.79 ± 4.53	9.65 ± 3.31	0.77
Vitamin B1 (mg/d)	1.91 ± 0.87	2.06 ± 0.77	0.32	2.30 ± 0.82	2.12 ± 0.86	0.06
Vitamin B2 (mg/d)	2.30 ± 1.06	2.20 ± 0.96	0.59	2.40 ± 1.06	2.30 ± 1.25	0.47
Vitamin B3 (mg/d)	21.59 ± 2.50	21.39 ± 2.57	0.67	21.61 ± 2.90	21.70 ± 3.06	0.81
Vitamin B6 (mg/d)	2.05 ± 0.83	1.87 ± 0.79	0.25	1.82 ± 0.77	1.99 ± 0.90	0.09
Folate (mcg/d)	528.61 ± 86.76	512.65 ± 99.73	0.34	574.39 ± 95.19	566.94 ± 68.20	0.48
Vitamin B12 (mcg/d)	4.53 ± 1.67	4.43 ± 2.06	0.75	4.31 ± 1.76	4.33 ± 2.92	0.94
Pantothenic acid (mg/d)	5.13 ± 1.53	5.71 ± 1.87	0.06	5.64 ± 1.65	5.22 ± 2.08	0.06
Biotin (mcg/d)	26.76 ± 3.75	27.02 ± 4.98	0.74	29.33 ± 6.61	28.40 ± 8.50	0.30
Vitamin C (mg/d)	146.20 ± 22.40	145.34 ± 20.56	0.83	154.33 ± 36.99	151.11 ± 67.55	0.60
Vitamin C (mg/d)	146.20 ± 22.40	145.34 ± 20.56	0.83	154.33 ± 36.99	151.11 ± 67.55	0.60
Vitamin D (mcg/d)	1.14 ± 0.82	0.99 ± 0.83	0.34	1.04 ± 0.77	1.02 ± 0.78	0.87
Vitamin K (mcg/d)	157.89 ± 30.40	162.51 ± 55.33	0.53	146.75 ± 21.63	143.89 ± 27.75	0.33

Logistic regression of the association between CRC and dietary intake among people with TT FTO rs9939609 genotype indicated no association between CRC risk and fiber intake (Table 4). The results did not change after adjusting for age and sex (model 2), additional adjustments for smoking, alcohol consumption, and physical activity (model 3), and further adjustments for BMI and calorie intake (Model 4).

**Table 4:** Logistic regression of the association between colorectal cancer and dietary intake among people with TT FTO rs9939609 genotype.

	**Model 1**		**Model 2**		**Model 3**		**Model 4**	
	**OR (CI 95%)**	***P***	**OR (CI 95%)**	***P***	**OR (CI 95%)**	***P***	**OR (CI 95%)**	***P***
Soluble fiber	0.90 (0.55–1.49)	0.69	0.85 (0.51–1.41)	0.53	0.85 (0.51–1.41)	0.54	0.85 (0.51–1.40)	0.52
Insoluble fiber	0.83 (0.67–1.03)	0.06	0.81 (0.65–1.01)	0.07	0.81 (0.65–1.01)	0.06	0.82 (0.66–1.03)	0.09
Crude fiber	0.97 (0.83–1.15)	0.76	0.95 (0.81–1.12)	0.56	0.95 (0.80–1.12)	0.52	0.95 (0.80–1.12)	0.55
Total fiber	0.99 (0.90–1.11)	0.94	1.02 (0.91–1.40)	0.74	1.02 (0.91–1.14)	0.71	1.02 (0.91–1.14)	0.75

Model 1, crude form, adjusting for age and sex; (model 2), adjusting for age, sex, smoking, alcohol consumption, and physical activity; (model 3), adjusting for age, sex, smoking, alcohol consumption, and physical activity, BMI, and calorie intake (Model 4).

The association between CRC and dietary fiber intake among people with AA/AT FTO rs9939609 genotype is presented in Table 5. An inverse association was found between CRC and total amounts of dietary fiber intake (OR: 0.91, CI 95%, 0.84–0.99, *p* = 0.03) among people with AA/AT FTO rs9939609 genotype. The association remained significant after adjusting for age and sex (model 2), additional adjustments for smoking, alcohol consumption, and physical activity (model 3), and further adjustments for BMI and calorie intake (model 4).

**Table 5:** Logistic regression of the association between colorectal cancer and dietary fiber intake among people with AA/AT FTO rs9939609 genotype.

	**Model 1**		**Model 2**		**Model 3**		**Model 4**	
	**OR (CI 95%)**	***P***	**OR (CI 95%)**	***P***	**OR (CI 95%)**	***P***	**OR (CI 95%)**	***P***
Soluble fiber	1.18 (0.68–2.02)	0.54	1.27 (0.73–2.22)	0.39	1.27 (0.72–2.22)	0.39	1.22 (0.069–2.16)	0.48
Insoluble fiber	1.16 (0.96–1.4)	0.12	1.14 (0.94–1.38)	0.17	1.14 (0.94–1.38)	0.17	1.16 (0.95–1.4)	0.13
Crude fiber	0.91 (0.79–1.04)	0.19	0.086 (0.77–1.03)	0.13	0.89 (0.77–1.03)	0.13	0.91 (0.79–1.05)	0.21
Total fiber	0.91 (0.84–0.99)	0.03	0.91 (0.83–0.99)	0.03	0.91 (0.83–0.99)	0.03	0.92 (0.84–0.90)	0.04

Model 1, crude form, after adjusting age and sex; (model 2), adjusting age, sex, smoking, alcohol consumption, and physical activity; (model 3), adjusting age, sex, smoking, alcohol consumption, and physical activity, BMI, and calorie intake (Model 4).

## Discussion

**Figure 1:**
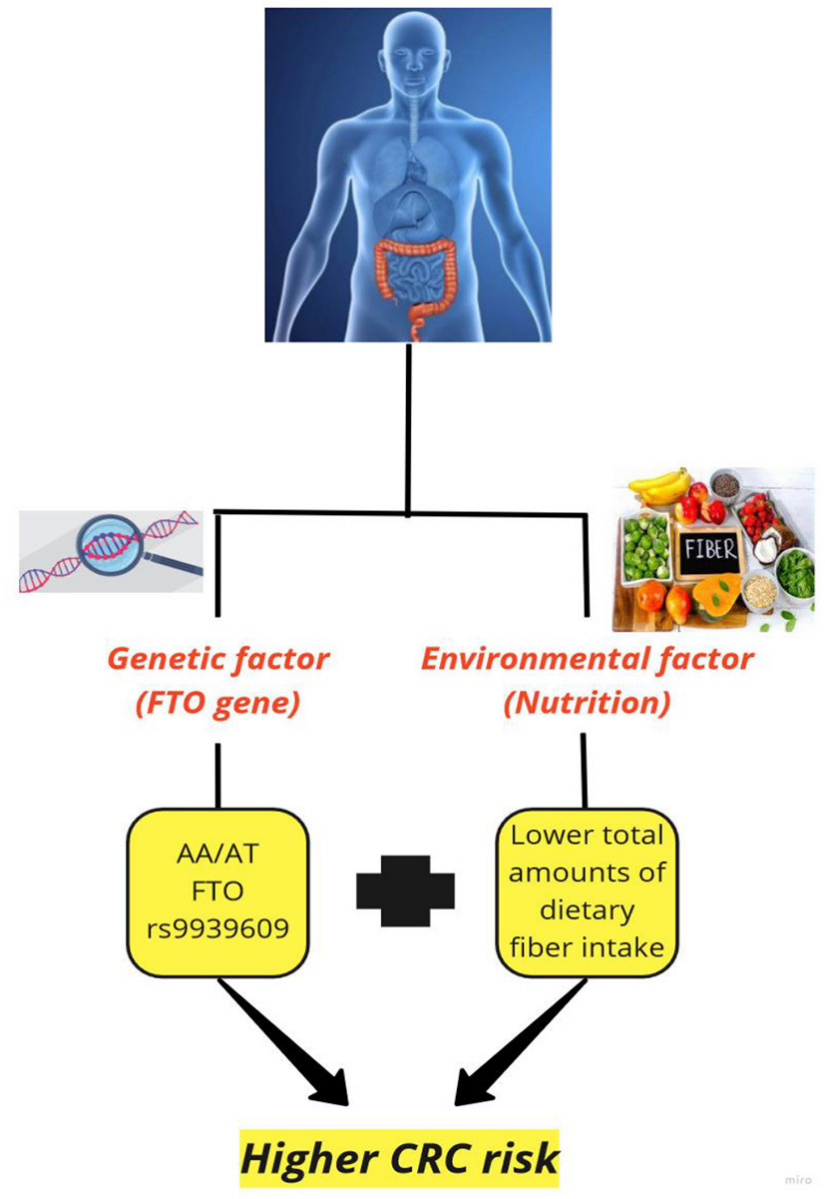
The effect of fat mass and obesity-associated (FTO) gene on the association between colorectal cancer and dietary fiber.

This study was among the first investigations on the effect of FTO genotype on the association between CRC and dietary fiber. The present case-control study demonstrated that CRC patients had a lower dietary fiber intake. Also, an inverse association was found between CRC and total dietary fiber intake only among people with AA/AT FTO rs9939609 genotype (Figure 1).

Few studies demonstrated the association of the FTO polymorphism with dietary fiber intake and obesity (20–22). In line with the present study, Koochakpoor et al. (20) showed that higher fiber intakes might decline obesity risk in risk allele carriers of FTO polymorphisms including rs17817449, rs8050136, and rs3751812. Hosseini-Esfahani et al. (21) reported that those with a high intake of dietary fiber and a higher number of risk alleles had a more pronounced effect of the FTO SNPs on general obesity. In addition, Czajkowski et al. (22) found that the effect of FTO SNPs on body composition and lipid profile might be modified by dietary fiber intake. According to their study, fiber intake above 18 gr/day for carriers of the GG genotype of rs3751812, CC genotype of rs8050136, and GG genotype of rs6499640 polymorphisms may positively affect anthropometric parameters, including a decrease in hip circumference.

A close connection was reported between FTO genotype and the risk of various cancers risk such as colon cancer (23). For example, one study found that rs1558902, rs8050136, rs3751812, and rs9939609 FTO SNPs had a positive association with colorectal cancer in Japanese population (23). Whereas, Yang et al. examined 677 FTO SNPs in patients from the colon cancer Family Registry and did not find any evidence that FTO SNPs were related to colorectal cancer risk (24).

Regarding to the association between CRC with obesity and the FTO gene, some studies reported that polymorphisms within the first intronic region of the FTO gene may exert their effects on CRC risk through regulation of the expression level of the FTO gene and subsequent modulation of leptin and adiponectin signaling (23, 24). Leptin may have a potential to directly stimulate cell proliferation and survival. And adiponectin has a potential to inhibit the leptin-induced signaling cascade (25). Lower adiponectin serum level was related to elevated risk of CRC among carriers of the FTO risk allele (26). Moreover, FTO gene upregulation may play a role in colorectal carcinoma cells through phosphatidylinositol-3-kinase (PI3K)/Akt/mammalian target of rapamycin (mTOR) pathway. Also, FTO gene overexpression may increase the serum level of leptin which may lead to the upregulation of pro-inflammatory genes such as IL6, IL1-beta, and enhanced VEGF-induced angiogenesis (19). FTO gene was reported to be a potential target gene for microRNA-1,266 which influence the proliferation of colorectal cancer cell lines (25). The microRNA-1,266 expression is negatively correlated to FTO gene and knockdown of microRNA-1,266 remarkably promoted the viability of CRC cells. Therefore, microRNA-1,266 may be a tumor-suppressor gene in CRC, which affects the progression of CRC *via* targeting FTO (25). *In vitro* experiments have shown that FTO has effective demethylation activity targeting N6-methyladenosine (M6A) sites in RNA and overexpression of FTO leads to a decrease in M6A in human cells (26). M6A is a ubiquitous RNA modification that plays a vital role in human tumors (27). A study showed that M6A-related genes were dysregulated in CRC, and their expression was associated with CRC progression and poor prognosis (28). Moreover, FTO gene may have a key role in mucosal immune health (27). A recent study found that m6A modification may affect immune infiltration and therapeutic response in inflammatory bowel disease (IBD) (28).

On the other hand, FTO gene polymorphisms can increase the risk of colorectal cancer through the effect on body weight (29). An epidemiological study found a strong association between obesity and CRC (30) and a 5 kg/m^2^ increase in BMI was reported to be associated with a 5% increase in CRC risk (31). Obesity-related disorders such as insulin resistance and hyperinsulinemia are associated with increased production of growth factors needed for tumorigenesis, contributing to carcinogenesis. Inflammatory processes (32) and oxidative stress (33) are other obesity-related complications leading to cancer. Furthermore, the higher intake of carbohydrates, fat, and calorie were significantly associated with FTO risk allele (13). It is possible that the tumorigenic effects of the FTO gene are caused by the effect of polymorphisms of this gene on increasing the intake of macronutrients.

In terms of the effect of dietary fiber on CRC, previous studies (34–37) demonstrated the beneficial effects of dietary fiber on reducing CRC risk. Dietary fibers are thought to have a protective role against cancer by the fermentation of fibers by gut bacteria and production of short-chain fatty acids (SCFAs) (34). Among different types of SCFA, butyrate has been extensively studied for its anti-proliferative effects against CRC (38). Butyrate inhibits neuropilin-1, a receptor of a vascular endothelial growth factor (VEGF) commonly found in CRC cells (39). Also, by regulating the expression of microRNAs such as miR-92a and miR-203, the butyrate may induce apoptosis and suppress the proliferation and invasion of CRC cells (40, 41). Furthermore, butyrate may inhibit the motility of CRC cells by inhibiting the Akt/ERK signaling pathway, indicating the potential of butyrate in blocking metastatic CRC (42). As a result, increased fiber consumption may mitigate cancer risk by increasing butyrate levels in the body (43). Recent studies reported that dietary fibers and butyrate may suppress tumorigenesis induced by the Akt pathway (44, 45). Activation of Akt signaling has been reported in 60–70% of human colon cancers and inhibitors of PI3K/Akt signaling have been suggested as potential therapeutic agents (46). Given that the FTO gene also plays a role in carcinogenesis through AKT signaling pathway, the interaction of the effects of dietary fiber and the FTO gene on this signaling pathway may be a crucial factor in CRC risk prevention (47).

However, this study had some limitations. First, the sample size was relatively small in the case group. Second, the FFQ was used to collect information about food intake, which is a self-report tool and its accuracy may be questioned. Third, the cases were selected from patients who had stages 3 and 4 CRC and underwent adjuvant chemotherapy with FOLFOX4 after surgery and the results cannot be generalized to other CRC patients.

## Conclusion

The present study found an inverse association between higher consumption of dietary fiber and a lower risk of CRC in risk allele carriers of FTO gene. Dietary fiber may play a role in preventing colon cancer in people with FTO gene risk allele. Further studies are needed to increase our understanding of the interaction between CRC, FTO gene, and fiber.

## Data availability statement

The original contributions presented in the study are included in the article/supplementary materials, further inquiries can be directed to the corresponding author.

## Ethics statement

This research follows the code IR.SBMU.CRC.REC.1398.028 on 12/11/2019 approved by the Research Ethics Committee of the Cancer Research Center of Shahid Beheshti University of Medical Sciences, Tehran, Iran. Written informed consent to participate in this study was provided by the participants.

## Author contributions

SD and MG designed the study and analyzed data. SF, MAh, MV, MAf, ZR, NH, SS, SP, SA, and EK cooperated in the implementation of the study. All authors listed have made a substantial, direct, and intellectual contribution to the work and approved it for publication.

## Conflict of interest

The authors declare that the research was conducted in the absence of any commercial or financial relationships that could be construed as a potential conflict of interest.

## Publisher's note

All claims expressed in this article are solely those of the authors and do not necessarily represent those of their affiliated organizations, or those of the publisher, the editors and the reviewers. Any product that may be evaluated in this article, or claim that may be made by its manufacturer, is not guaranteed or endorsed by the publisher.
